# Brazilian Strategy for Breastfeeding and Complementary Feeding Promotion: A Program Impact Pathway Analysis

**DOI:** 10.3390/ijerph19169839

**Published:** 2022-08-10

**Authors:** Daiane Melo, Sonia Venancio, Gabriela Buccini

**Affiliations:** 1 Department of Nutrition, School of Public Health, University of São Paulo, Professor Mello de Moraes Avenue, 1235, São Paulo 05508-030, SP, Brazil; 2 Health Institute of São Paulo, State Health Secretariat, R. Santo Antônio, 590, Bela Vista, São Paulo 01314-000, SP, Brazil; 3 Department of Social and Behavioral Health, School of Public Health, University of Nevada Las Vegas, Las Vegas, NV 89119, USA

**Keywords:** breastfeeding, complementary feeding, health policy, primary health care, implementation analysis, Program Impact Pathway

## Abstract

Background: The Brazilian Breastfeeding and Complementary Feeding Strategy (Estratégia Amamenta e Alimenta Brasil—EAAB) aims to improve Primary Health Care (PHC) workers’ counseling skills to promote and support infant and young children feeding (IYCF). However, the maintenance and scaling up of the EAAB has been challenging. The theory-driven Program Impact Pathway (PIP) is recommended to assess and enhance the large-scale implementation of IYCF programs. The purpose of this study was to document barriers and facilitators to scale up the EAAB using a PIP analysis. Methods: First, we reviewed EAAB documents to develop an initial PIP diagram. Then, we interviewed EAAB key informants to identify Critical Quality Control Points (CCP) in the PIP. We revised and analyzed the PIP to inform the EAAB core functions and pathways. Results: Six CCPs for EAAB maintenance were identified: CCP1—Definition and strengthening of the coordination in states and municipalities; CCP2—Maintenance of tutors’ work; CCP3—Feasibility of the certification process; CCP 4—Quality improvement of IYCF activities in PHC units; CCP 5—Adequate use of monitoring systems; and CCP 6—Consistent implementation monitoring. Four implementation pathways and seven core functions identified may assist with scaling up the EAAB in Brazil. Conclusion: The PIP analysis proved to be useful for documenting the factors that influence the maintenance and scaling up of the EAAB.

## 1. Introduction

Implementing policies, services, and interventions is essential for promoting, protecting, and supporting early childhood development. In this context, the global Nurturing Care Framework (NCF) outlines five components that must be taken into consideration to help children develop and thrive through the lens of equity, integrity, and social justice. Adequate nutrition is one of the components of NCF, and includes breastfeeding (BF) and healthy complementary feeding (CF). It is well established that BF and healthy CF are critical for promoting children’s health and optimum development in the first 1000 days of life [1,2]. Furthermore, BF also has a significant impact on women’s health by reducing the risk of breast cancer and type 2 diabetes mellitus [3]. BF is also positively associated with intelligence test performance in childhood and adolescence, and thus contributes to child development [4] and the achievement of the 2030 Sustainable Development Goals [1].

Although breastfeeding has advantages for both mothers and their children, breastfeeding rates are not optimal, globally [5]. In 2020, only 44% of children were exclusively breastfed in the first six months of life, far below the global Maternal, Infant, and Young Child Nutrition target to reach at least 70% exclusive BF by 2030 [6]. Improving CF is also urgent, as only 29% of children receive the minimum dietary diversity in the first year of life [6]. Data from 57 low- and middle-income countries (LMICs) showed worrying global weighted prevalence during 2010–2018: 45.7% for exclusive breastfeeding under 6 months, and 32.0% for exclusive breastfeeding at 4–5 months. In this same study, the prevalence of the introduction of solid, semi-solid, or soft foods under 6 months were 14.9% and 63.1% at 6–8 months [5].

Brazil, the largest country in Latin America and the Caribbean, has been implementing large-scale infant and young child feeding (IYCF) policies and programs, which has improved exclusive BF prevalence from 3.1% (in 1986) [7] to 41.3% (in 2008) [8]. However, a great proportion of children (80.5%) between 6 and 23 months of age consumed ultra-processed and unhealthy foods in 2019 [7]. A national study showed that the exclusive BF prevalence trend in Brazil slowed down between 2006 and 2013 [9]. In 2019, about 45.8% of children under six months were exclusively breastfed [10]. Therefore, Brazil is unlikely to meet the 2030 targets unless sustainable investments in scaling up effective programs are made [6].

Strong evidence supports the idea that counseling interventions targeting pregnant women and mothers with young children are key for supporting women in initiating and continuing BF and to improving CF, especially community-based interventions, including group counselling or education and social mobilisation [3,11,12]. It is important that healthcare workers are trained to support mothers and children to overcome breastfeeding difficulties and to adopt optimal feeding practices [3,11]. This interaction and follow-up is essential for mothers to continue breastfeeding, even in the face of difficulties at the individual level, such as diminished maternal confidence and self-efficacy, inadequate breastfeeding positioning, and biological factors like the anatomy of the breast, nipple pain and fissure, grip, and changes in the newborn’s mouth anatomy, such as ankyloglossia [3,11,13]. Social challenges are also relevant, and proper counseling can help mothers make the decision to continue breastfeeding even with the influences of family and partners, and the challenges of returning to work [3,11].

The Brazilian Ministry of Health (MoH) recommends the implementation of the Brazilian Breastfeeding and Complementary Feeding Strategy (Estratégia Amamenta e Alimenta Brasil—EAAB) to improve the counseling skills of primary health care (PHC) workers regarding the promotion and support of IYCF practices [14]. The EAAB is fully described in Table S1, according to the Template for Intervention Description and Replication (TIDieR) [15]. The EAAB is grounded on a critical and reflective thinking methodology and proposes a continuing learning process led by its tutors. EAAB tutors work as coaches, organizing workshops at PHC units to improve PHC workers’ counseling skills to support and promote BF and healthy CF [16,17]. A recent literature review on what works to protect, promote, and support breastfeeding on a large scale highlighted the importance of continuity of care and support in community and family settings via home visits delivered by community health workers, and community involvement [18]. In accordance with this, as part of implementation of the EAAB, PHC workers discuss in groups how to organize an action plan to provide adequate nutritional care for mothers and children in the community. The EAAB is managed at the federal, state, and municipal levels, and coordinators are in charge of planning, funding, and monitoring its implementation in PHC units. An implementation certification is given by the MoH to the PHC units that meet six criteria, which are important criteria for realizing the impacts of the EAAB [14,19].

Scaling up and maintaining the EAAB is challenging because Brazil has continental dimensions and great socioeconomic and demographic disparities. In 2016, only 9.4% out of the 42,000 Brazilian PHC units implemented the EAAB [20,21]. In this scenario, some progress was achieved; in 2019, more than 5900 EAAB tutors were trained and 48,640 PHC workers participated in at least one EAAB workshop. However, only 192 PHC units were certified, i.e., fulfilled the six EAAB criteria [22]. The MoH commissioned a national project to support the EAAB scale-up that included an implementation analysis in two states. This analysis was carried out using the Program Impact Pathways (PIP) to understand the program theory and the barriers to and facilitators of the maintenance and scaling up of the EAAB.

Theory-driven PIP analyses have proven useful for mapping the mediating processes between program inputs and outcomes in child nutrition and development initiatives [23,24]. Thus, a PIP analysis is key for assessing the implementation of the program and the contextual factors that might affect the intervention when scaling up a program in complex settings, as is the case of the EAAB in Brazil [24,25]. The hypothesis of this study is that understanding the EAAB program theory with a PIP analysis will help identify which are the essential activities for maintaining and scaling up the EAAB [23,24,25]. Hence, this study aimed to document barriers to and facilitators of implementation of the EAAB in Brazil through a PIP analysis.

## 2. Materials and Methods

**Figure 1:**
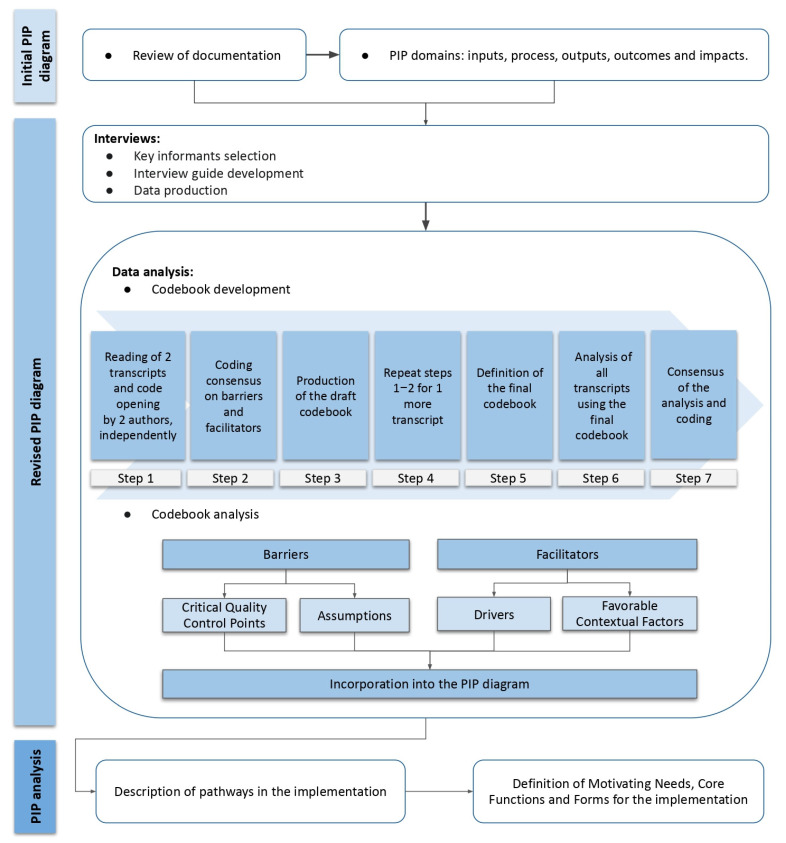
Methodological steps of PIP analysis to document implementation of the EAAB. Source: Developed by the authors. Notes: EAAB (Brazilian Breastfeeding and Complementary Feeding Strategy (Estratégia Amamenta e Alimenta Brasil)); PIP (Program Impact Pathway).

This is an exploratory qualitative study based on the PIP analysis proof of concept to document implementation of the EAAB [23,26]. The methodological steps are described in Figure 1. First, an initial PIP diagram was developed based on an EAAB document review. Second, barriers and facilitators were identified through semi-structured interviews with EAAB key informants (KIs). Barriers were considered any difficulty or challenge related to the implementation and scaling up of the EAAB [27]. Facilitators were considered factors contributing positively to its implementation and strategies that help deal with the barriers identified [27]. Findings were incorporated into the revised PIP diagram and informed the Critical Quality Control Points (CCP) for maintenance of the EAAB. Third, a PIP analysis identified core functions and pathways for scaling up of the EAAB.

### 2.1. Initial PIP Diagram

#### 2.1.1. Review of Documentation

A review of the Brazilian MoH guidelines and publications related to implementation of the EAAB was carried out. This review included the following documents: the EAAB Implementation Guide [16], the Protocol for the EAAB Implementation [28], and the Joint Technical Note on the EAAB certification process for the year 2015 [29].

#### 2.1.2. PIP Domains

An initial PIP diagram with five domains was developed based on EAAB document review, including multilevel activities developed by federal, state, and municipal stakeholders for implementation of the EAAB. Table 1 summarizes the operational definitions of the PIP domains: (1) inputs, (2) processes, (3) outputs, (4) outcomes, and (5) impacts.

### 2.2. Revised PIP Diagram

#### Interviews

Key informants’ selection: we interviewed eight KIs based on their unique experiences with the implementation of the EAAB at the federal (*n* = 3), state (*n* = 2), and municipal levels (*n* = 3). Two KIs that worked at the federal level organized the national implementation of the EAAB and worked in the MoH Coordination of Food and Nutrition and Coordination of Child Health and Breastfeeding. One KI was a national trainer of the International Baby Food Action Network (IBFAN), which supported the MoH in the EAAB training workshops. The KIs that worked at the state level were chosen based on their experience as EAAB state coordinators. First, we selected two states based on the proportion of PHC units that joined the EAAB, according to MoH’s administrative data. The Federal District, located in the Center-West of Brazil, was selected for having the highest proportion of PHC units implementing the EAAB, while São Paulo, located in the Southeast, was selected for having the lowest proportion. We selected three KIs that worked at the municipal level from two municipalities in the state of São Paulo (Embu das Artes and Ribeirão Preto). Two of them worked as municipal coordinators and one of them as a tutor of the EAAB. These KIs had important experience in obtaining the EAAB certification in the state, which has one of the lowest adoption rates.

Interview guide development: the interview guide was drafted based on the document review and the initial PIP diagram. Questions assessed barriers to and facilitators of the implementation of the EAAB [30,31]. A pilot interview was carried out with a professional that coordinates the EAAB in a state not included in this study (Mato Grosso). Adjustments to the interview guide were made to adapt the questions to each participant’s experience and regional context (i.e., federal, state, and municipal levels).

Data production: semi-structured interviews were conducted in person (*n* = 1) or virtually (*n* = 7) from December 2019 to April 2020 and lasted about 60 min. Paraphrasing was used when necessary to ensure that the interpretation of the participants’ responses was accurate. The interviews were audio-recorded with permission, verbatim transcribed, and then checked for accuracy.

### 2.3. Data Analysis

#### 2.3.1. Codebook Development

We used seven systematic steps to conduct a thematic analysis of the transcripts (Figure 1) [30]. This approach has been widely used in studies investigating the implementation of IYCF programs [25,32,33]. In the analysis, we interpreted the KIs’ perceptions about the implementation of the EAAB to define the barriers and facilitators [30,31], i.e., the characteristics that disrupt or enhance the dissemination and implementation of the EAAB [27]. The transcripts were coded in barriers and facilitators based on deductive and inductive approaches. We created a spreadsheet structured around the five domains of the PIP diagram [30,31]. Then, we opened the codes within each of these domains, coding two transcripts independently. We reviewed and discussed the coded transcripts to reach a consensus and created a draft codebook, which was used to code one more transcript independently. We then defined the final codebook, which was used to code the remaining five interviews. The codebook was interactively revised and vetted multiple times with the help of the research team to ensure transparency and agreement on code organization. Lastly, the quotes that most accurately represented barriers and facilitators were translated from Portuguese into English.

#### 2.3.2. Codebook Analysis

Barriers were summarized in the codebook as Critical Quality Control Points (CCP) (i.e., any activity that requires systematic monitoring to maximize the quality, effectiveness, and maintenance of the implementation) or Assumptions (i.e., essential activities for EAAB sustainability). Facilitators were summarized as Drivers (i.e., factors that directly facilitate the implementation of the EAAB) or Favorable Contextual Factors (i.e., conditions or external contexts that support and positively influence EAAB activities).

### 2.4. PIP Analysis

Guided by the revised PIP, we described four pathways in which the EAAB has been implemented, highlighting barriers or facilitators that modulated the success of the implementation scaling up. Lastly, we followed the framework proposed by Jolles et al. [34] to identify the Motivating Needs, Core Functions, and Forms in the revised PIP, describing, this way, the essential constructs to achieve a successful implementation. These constructs are defined as follows: (a) Motivating Needs—the ignition to the development of an intervention; (b) Core Functions—a set of intended structural and procedural goals that can guide the fidelity assessment of a program; and (c) Forms—specific steps and activities taken to perform each Core Function that may be customized to local contexts [34].

## 3. Results

### 3.1. Initial PIP Diagram

The initial PIP diagram summarizes each stage of the implementation of the EAAB (Figure S1). In brief, the implementation of the EAAB starts with “inputs,” activities developed at the federal level, such as funding, monitoring systems management, and establishing a group of national trainers. These elements connect with “processes,” activities developed in states and municipalities. Professionals from Health Secretariats are responsible for coordinating and funding the EAAB, as well as organizing training workshops and monitoring and disseminating outcomes. The expected “output” of these activities is that PHC workers fulfill the six criteria for implementation certification. The training workshops and PHC units certification lead to the EAAB “outcomes,” i.e., improvement of the PHC workers’ skills to promote BF and healthy CF. Finally, the interventions led by PHC workers can positively affect BF and CF indicators, as well as children’s nutritional profiles.

### 3.2. Revised PIP

**Figure 2:**
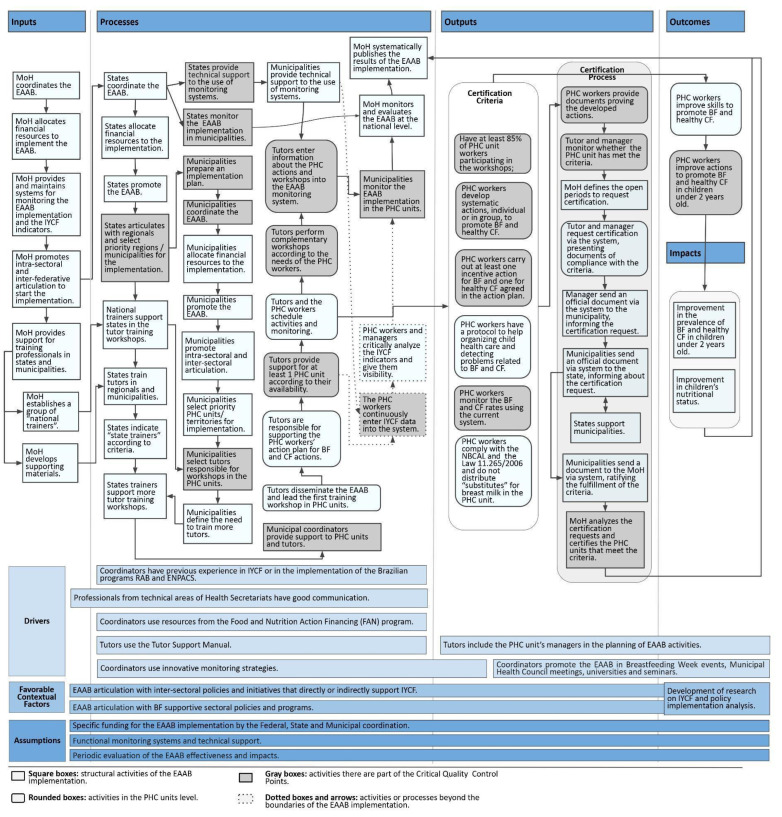
Revised PIP diagram of the implementation of the EAAB. Source: Developed by the authors with data obtained from document review and interviews with key informants. Notes: BF (breastfeeding); CF (complementary feeding); EAAB (Brazilian Breastfeeding and Complementary Feeding Strategy (Estratégia Amamenta e Alimenta Brasil)); ENPACS (National Strategy for Promotion of Complementary Healthy Eating (Estratégia Nacional para Alimentação Complementar Saudável)); IYCF (Infant and Young Child Feeding); MoH (Ministry of Health); NBCAL (Brazilian Norm for the Commercialization of Food for Infants and Young Children, Pacifiers, and Baby Bottles (Norma Brasileira de Comercialização de Alimentos para Lactentes e Crianças de Primeira Infância, Bicos, Chupetas e Mamadeiras)); PHC (Primary Health Care); RAB (Brazilian Breastfeeding Network (Rede Amamenta Brasil)).

We identified six CCPs in the EAAB revised PIP (Figure 2):

CCP1—Definition and strengthening of the coordination in states and municipalities (e.g., lack of political formalization of a municipal coordinator);

CCP2—Maintenance of tutors’ work (e.g., difficulty with identifying professionals with an adequate profile and workload availability to be a tutor);

CCP3—Feasibility of the certification process (e.g., difficulty with fulfilling four of the six criteria and delayed certification process due to lack of system integration);

CCP4—Quality improvement of IYCF activities in PHC units (e.g., difficulty with keeping PHC workers updated due to high turnover);

CCP5—Adequate use of monitoring systems (e.g., low-quality data about the IYCF interventions developed in PHC units and low coverage on the IYCF indicators);

CCP6—Consistent monitoring of the implementation (e.g., irregular monitoring of the certified PHC units).

In addition, specific funding for the EAAB, functional monitoring systems, and periodic EAAB evaluation were identified as Assumptions for the sustainability of the implementation (Figure 2). Table 2 describes the CCPs and Assumptions.

In Table 3, we summarized the Drivers and Favorable Contextual Factors and aspects that positively influence the implementation of the EAAB, such as engaged municipal coordinators, good articulation between technical areas at the federal, state, regional, and municipal levels, and presence of inter-sectoral policies (including BF initiatives and programs).

### 3.3. PIP Analysis

We identified four EAAB implementation pathways and highlighted the facilitators and barriers for implementation of the EAAB (CCPs, Assumptions, Drivers, and Favorable Contextual Factors). The PIP analysis also informed seven Core Functions to guide the successful implementation of the EAAB in large scale (Table 4). Some Core Functions were highlighted in the description of the EAAB implementation pathways, informing if they were being implemented or not.

#### 3.3.1. Pathway for Financing and Scaling Up the EAAB

The MoH coordinated the EAAB and articulated the initiation of EAAB activities in the states (Core Function implemented). The EAAB did not receive specific funding (Core Function not implemented), but the MoH transferred resources to states and municipalities through the Food and Nutrition Fund (FAN) to support the implementation of actions and health services focused on this theme (Driver). Then, states and municipalities may use the FAN resources or allocate their own resources to implement the EAAB. In general, KIs believed that creating specific funding for the EAAB would be important to sustain the training workshops (Assumption). In addition, some state coordinators communicated with regional health departments that helped expand the EAAB to municipalities (Driver). The state coordination developed an implementation plan, as recommended in the EAAB implementation guide (Core Function implemented) [16]. However, in the municipalities, there was not a politically formalized municipal coordinator, neither there was a municipal plan for the implementation of the EAAB (CCP 1). In contrast, when the municipal coordinator was engaged with the EAAB (Driver), they helped to establish intra-sectoral partnerships that supported the implementation of the EAAB.

#### 3.3.2. Pathway for Improving Professional Skills

The MoH provided theoretical materials and established a group of national trainers to lead tutor training workshops within the states. The state coordinators appointed state trainers to lead more tutor training workshops (Core Function implemented). The municipal coordinators selected tutors to join the training workshops, although sometimes they had difficulty finding suitable professionals (CCP 2). KIs stated that, in many municipalities, tutors were unable to give training workshops in various PHC units (CCP 1) due to lack of assistance from the coordinator. As a result, many tutors gave up tutoring (CCP 2). Tutors were expected to continuously support PHC workers by leading complementary training workshops so that they could design activities to support and promote IYCF and help obtain the implementation certification (Core Function not fully implemented). The high turnover of tutors and PHC workers, however, posed a significant barrier to achieving these outcomes (CCP 4).

#### 3.3.3. Pathways for Certification

To request the implementation certification, PHC workers had to participate in the first training workshop, develop individual and group activities with mothers and families with children from 0 to 2 years old (such as counseling sessions and prenatal/postnatal educational groups), define a protocol to organize child health care, monitor the IYCF indicators using the system, and should not have distributed breastmilk substitutes in the PHC unit, complying with Brazilian law (Core Function not fully implemented). Briefly, to request certification via the system, tutors and PHC unit managers had to send supporting documents to the MoH, which were verified by the state coordinator to assure they met all the certification criteria. The requests were evaluated by the MoH to generate the certification. The KIs reported many barriers to complying with the certification criteria (CCP 3) and system instabilities, which delayed the evaluation of certification requests by the MoH (CCP 5). Despite the challenges, some PHC units in the states and municipalities were accredited in the EAAB.

#### 3.3.4. Pathways for Monitoring and Evaluating

The MoH provided the systems to monitor the training workshops and the IYCF indicators, and state and municipal coordinators had to guide tutors and PHC workers to properly enter data into the system (Core Function not fully implemented/Assumption). Several problems in accessing and feeding these systems were reported, although municipal coordinators used some complementary monitoring strategies that improved tracking the activities, such as regular meetings with tutors and contact with tutors via WhatsApp groups (Driver). The MoH examined certification requests in the systems and was supposed to share the outcomes of the implementation of the EAAB regularly (Core Function not fully implemented/Assumption). However, there was a gap in implementation monitoring and evaluation due to a lack of user-friendly monitoring systems, low coverage of IYCF indicators, poor data quality entered into the systems, and the numerous PHC units to be evaluated (CCP 5). Also, the maintenance of the PHC unit certification (CCP 6) and the impact of EAAB on IYCF indicators were not assessed (Assumption).

## 4. Discussion

Our study systematically analyzed barriers to and facilitators of the implementation of the EAAB, proving the concept that PIP analysis is useful for identifying mediators who can shape the maintenance and scaling up of the EAAB in Brazil [26]. Therefore, PIP analysis might further be used in expanded implementation research of the EAAB in other regions of Brazil. This study’s results can help EAAB stakeholders understand barriers and facilitators at the three implementation levels, and can provide implementation quality indicators for CCP, Assumptions, and Core Functions [24,34,35].

The four EAAB implementation pathways highlighting the CCP, Assumptions, Drivers, and Core Functions helped to explain why some activities favored or challenged the success of the implementation. It is important to note that the EAAB results from the process of revising and analyzing the PIP are connected and complement each other. For example, the Core Functions encompass the Assumptions identified in the revised PIP, as both include aspects that are fundamental to the execution of EAAB in the long term. Another relevant result is that the CCP’s cover activities are also part of the EAAB Core Functions, and they should be monitored to guarantee the maintenance of the quality of the implementation.

We identified funding as an Assumption and also as one of the Core Functions for the sustainability and successful implementation of the EAAB. Investing in the EAAB can be a significant step towards strengthening IYCF actions in PHC [3]. There is evidence that funding is critical for motivating the implementation of training-based programs for IYCF counseling [25,36]. In 2020, the MoH released an ordinance of cash incentives to 382 municipalities that had EAAB certifications. These municipalities are supposed to promote and support IYCF in PHC and to monitor child nutrition status, which is an advance for implementation of the EAAB, as it may help engage state and municipal EAAB coordinators, tutors, and PHC workers to develop EAAB actions. Future case studies of these municipalities might be useful to understand whether cash incentives helped the implementation and maintenance of the EAAB.

Defining formal coordination in municipalities is also part of the EAAB Core Functions and is a CCP for implementation maintenance. Previous evaluations of Brazilian strategies to improve IYCF in PHC settings showed that lack of formal coordination has been linked to unplanned and fidelity-inconsistent adaptations [37,38,39]. Evidence has indicated that, to scale up IYCF programs, coordination and monitoring should be the master gears directing the implementation and enabling the synchrony of the other elements (e.g., funding, professional training, program delivery, research, and evaluation) [40]. The Happy Child Program (Programa Criança Feliz), a Brazilian intersectoral nurturing care program, for example, scaled up quickly when states recruited a coordination team to provide technical support to municipalities [35]. Therefore, defining formal municipal coordination for EAAB is crucial to strengthen the commitment to its implementation.

Although the EAAB proposes an organized plan to train tutors and workers in PHC, the high turnover of these professionals is a great challenge to reaching the desired outcomes. This barrier has been frequently noted in the large-scale implementation of IYCF programs in low- and middle-income countries [35,40]. Providing cash incentives based on performance has been noted as a potential strategy to retain and motivate human resources [35,40]. In the context of the EAAB, a cash incentive strategy has not yet been implemented and might require continuous funding from the MoH to enact its implementation. Therefore, future studies may assess what strategies are feasible and effective to improve the retaining of tutors and health workers in order to develop EAAB actions.

In this context, continuous training in PHC units is important to train new workers and to follow up the improvement on the actions to promote BF and healthy CF. A manual for guiding tutors in the development of follow-up training workshops in PHC units proved to be an excellent tool to enhance tutors’ performance [14]. There is evidence that a minimum of monthly refresh training sessions improves IYCF counseling-based programs [36]. Therefore, it is relevant to closely monitor and incentivize these activities for the EAAB. Additionally, professional training through digital eHealth or mHealth is an effective strategy in high-, middle-, and low-income regions [41]. In this aspect, progress in the implementation of the EAAB has already been achieved, as the MoH launched two virtual courses in 2020 and in 2021 to support IYCF counseling, base on the Brazilian Guide for infant feeding [42], and to train tutors for the EAAB, respectively.

Regarding EAAB certification, it should work as an incentive for PHC workers to fully implement the EAAB, nevertheless, in practice, it is challenging to accomplish all the required criteria. Furthermore, we found that PHC workers developed actions to obtain the certification, but these activities were not sustained in the long term and have not improved BF and CF practices [14]. Similarly, in the Baby-friendly Hospital case, it is also difficult to maintain the certified institution designation [43]. On the other hand, specialists argue that the certification strategy can be useful to achieve large-scale implementation fidelity [40]. In the case of the EAAB, it might be necessary to review the feasibility of the certification criteria to guarantee the quality of implementation, taking into account the Core Functions identified in our study.

Finally, regular monitoring and frequent assessment of system performance are assumptions for successful large-scale implementation. However, monitoring the quality of health care programs is a challenge worldwide [44]. In Brazil, the IYCF surveillance system (Sisvan) covered only 5% of the national population in 2019 [45]. Due to the low coverage and few studies conducted to evaluate the impact of the EAAB [14,46], it is difficult to know the effectiveness of the EAAB in improving IYCF knowledge among health care workers and in improving the prevalence of BF and CF. As an IYCF supportive program, the EAAB should be evaluated at least every five years to increase the commitment to infant nutrition and to meet the Maternal and Early Childhood Nutrition targets for 2030 [47].

Consistent with prior analyses of complex adaptive systems, we can say that the CCP, assumptions, and drivers found in the EAAB PIP review jointly create complex phenomena that modulate the scaling up of the EAAB [48,49]. For example, the decision from state and municipality coordinators to designate funding for the implementation of the EAAB can be described as a path-dependence phenomenon, which means, this is a non-reversible adaptation that can lead to different outcomes [49]. Another illustration of complex phenomena in the implementation of the EAAB was feedback loops (i.e., when the output of a process within the system was fed back as an input into the same system) [49]. Coordinators periodically reporting the results of the implementation of the EAAB to Health Secretariats configured a positive feedback loop, as it increased the likelihood of the state or municipality allocating new funding to the EAAB. In contrast, a negative feedback loop happened when there was no formal municipal coordination for the EAAB, because it limited the support to tutors’ work. Therefore, future national analysis of the implementation of the EAAB may contribute to understand how effective scaling up can be done while considering the existing modulating complex phenomena, as well as the adaptations needed in different contexts [48,49].

We acknowledge that our study has some limitations. We interviewed a few KIs, which represented two states and two municipalities with engaged EAAB teams. These characteristics affect the external validation of our findings, as we might find different barriers and facilitators in other regions of Brazil. Future validation of the PIP diagram, including stakeholders from other Brazilian states and PHC workers, is planned, to understand the influence of the diversity of local context mediators and adaptations on EAAB implementation outcomes.

Several lessons were learned through the PIP analysis of the implementation of the EAAB. First, establishing exclusive funding for the EAAB at the federal, state, and municipal level is critical. In addition, the use of conceptual frameworks should be encouraged in resource-limited settings to help EAAB coordinators plan costs and expenses [50,51]. Second, formally assigning a municipal coordinator would strengthen scaling up of the EAAB, as their role is critical in assisting and motivating tutors in PHC units [39]. Third, incorporating innovative training strategies—such as the online training in IYCF counseling and the use of the Tutors Support Manual—may improve the performance of professionals [14,47]. Fourth, reviewing the certification criteria based on the Core Functions may benefit the quality and sustainability of the EAAB [34]. Finally, making the monitoring system more user-friendly would help the routine assessment of the quality of the EAAB [44,47].

## 5. Conclusions

In conclusion, the PIP analysis proved to be useful to documented barriers to and facilitators of the implementation of the EAAB, and brought to light Core Functions and implementation pathways relevant to its maintenance and scaling up. Multilevel coordination and EAAB activities have the potential to improve PHC workers’ counseling skills to promote and support good practices in IYCF. It is urgent to improve EAAB monitoring and invest in impact evaluation. Additionally, it is necessary for EAAB stakeholders to take into account the complex phenomena that modulate the scaling up of the EAAB, and to review some concepts relating to its implementation pathways. This implementation study contributes to the replication of the PIP analysis methodology in other countries, in order to study factors that influence the implementation of feeding and child nutrition programs, and to intervene when necessary.

## Figures and Tables

**Table 1:** Operational definition of the PIP diagram domains.

Domain	Operational Definition
Inputs	Activities that need to be in place at the federal level to start implementation of the EAAB.
Processes	Activities related to coordination at federal, state, and municipal levels, aiming at professional training and implementation monitoring.
Outputs	Expected results from the “process” domain activities; mechanisms by which the EAAB affects the IYCF counseling actions in PHC units and provides the implementation certification to PHC units.
Outcomes	Final results from the activities in the “process” and “outputs” domains.
Impacts	Impacts on the prevalence of BF and CF indicators and results on the nutritional status of children under 2 years old.

Source: definitions adapted from BUCCINI et al. [23]. Notes: BF (breastfeeding); CF (complementary feeding); EAAB (Brazilian Breastfeeding and Complementary Feeding Strategy (Estratégia Amamenta e Alimenta Brasil)); IYCF (Infant and Young Child Feeding); PHC (Primary Health Care).

**Table 2:** Critical Quality Control Points and Assumptions based on the barriers in the implementation of the EAAB.

Critical Quality Control Point	Challenges
CCP 1—Definition and strengthening of the coordination in states and municipalities	Lack of political formalization of a municipal coordinator; Lack of a municipal plan for the implementation of the EAAB; Lack of support from coordinators to tutors; Unclear communication between state and municipal coordinators.
CCP 2—Maintenance of tutors work.	Difficulty with identifying professionals with an adequate profile and workload availability to be a tutor; The difficulty of tutors maintaining long term work due to overlapping functions and conflict of goals; The difficulty of tutors with providing support to more than one PHC unit.
CCP 3—Feasibility of certification process.	Difficulty with achieving four of the six criteria * because of: a high demand for work in PHC units; a high turnover of PHC workers; tutors giving up tutoring PHC units for the long term; only occasional interventions to promote IYCF; a low recording of data on IYCF indicators in the monitoring system. PHC units that do not submit a certification request; Difficulties with organizing the supporting documents necessary to request certification; Instability and lack of integration in the systems; Delayed analysis of requests for certification by the MoH.
CCP 4—Quality improvement of IYCF activities in PHC units	Difficulty with keeping PHC workers updated due to a high turnover; A high turnover of tutors, so the training workshops have not continued; Difficulties with fulfilling the certification criteria impacted the quality of activities.
CCP 5—Adequate use of monitoring system.	Lack of maintenance of the EAAB implementation monitoring system and the Food and Nutrition Surveillance system (Sisvan); Instabilities in the systems used for data recording and for analyzing certification requests; Lack of detailed information about the activities developed in PHC units to record IYCF in the systems; Low coverage of data about IYCF indicators.
CCP 6—Consistent implementation monitoring.	Irregular monitoring of certified PHC units.
**Assumption for EAAB sustainability**	**Challenges**
Specific funding for the implementation of the EAAB	Lack of a specific funding transfer from MoH, states, or municipalities for the implementation of the EAAB; Lack of funding necessary to implement the EAAB, which was a major concern for municipal coordinators.
Functional monitoring systems and technical support	Constant instabilities in accessing the monitoring systems; Lack of support on how to use the systems.
Periodic evaluation of the EAAB effectiveness and impacts	Lack of monitoring to understand the type of activities carried out in PHC units and their effectiveness; Lack of an official evaluation of the impact on the knowledge and practices of mothers and caregivers on IYCF.

* Certification criteria with CCP: (1) Participation of at least 85% of PHC workers in the workshops; (2) Development of individual or group systematic actions to promote BF and healthy CF; (3) Compliance with at least 1 BF incentive action and 1 healthy CF action agreed in the PHC unit action plan; (4) Monitoring of BF and CF rates by the current system. Source: Produced by the authors. Notes: BF (breastfeeding); CF (complementary feeding); EAAB (Brazilian Breastfeeding and Complementary Feeding Strategy (Estratégia Amamenta e Alimenta Brasil)); IYCF (Infant and Young Child Feeding); MoH (Ministry of Health); PHC (Primary Health Care).

**Table 3:** Drivers and Favorable Contextual Factors based on the facilitators in the implementation of the EAAB.

Drivers	Opportunities
Coordinators have previous experience in IYCF or in the implementation of RAB and ENPACS	Previous work experience with IYCF can positively influence the interest of coordinators regarding the implementation of the EAAB; Previous work experience with the implementation of the Brazilian programs RAB and ENPACS can positively influence the engagement of coordinators with the implementation of the EAAB.
Professionals from technical areas of Health Secretariats have good communication	Good communication among professionals from technical areas of the Health Secretariat, such as the Child Health, the Food and Nutrition, and the Primary Health Care, facilitated coordination of the EAAB; Good communication was a driver to prioritize the EAAB in the government plan and to allocate funding to the implementation of the EAAB; The articulation between state coordinators with the regional Health Secretariats facilitated the scaling up of the EAAB to municipalities.
Coordinators use resources from the Food and Nutrition Action Financing Program (FAN)	The MoH annually transfers the FAN resources to the states and municipalities; The FAN is designed to fund activities on the Food and Nutrition agenda; The EAAB coordinators can use part of the FAN resources to support the EAAB training workshops.
Tutors use the Tutor Support Manual	The use of the EAAB Tutor Support Manual improved the performance of tutors leading complementary training workshops in PHC units; The Tutor Support Manual was developed by Relvas et al. [10] to guide tutors in their activities in PHC units and to give recommendations on how to conduct complementary workshops.
Tutors include PHC units’ managers in the planning of EAAB activities	Support from the PHC unit manager to the tutor was crucial for scheduling the training workshops and IYCF interventions; Good results were observed when tutors supported ongoing IYCF counseling activities in PHC units.
Coordinators use innovative monitoring strategies	Coordinators who followed tutors more closely through regular meetings improved their work and the implementation of the EAAB; The use of specific monitoring forms helped to collect data on the implementation of the EAAB; The use of the WhatsApp application helped coordinators follow up on tutors’ activities.
Coordinators promote the EAAB in Breastfeeding Week events, Municipal Health Council meetings, universities, and seminars	Publicizing the EAAB in events and the municipal Health Council helped PHC units’ managers to learn about the benefits of implementing the EAAB and why they should invest in it.
**Favorable Contextual Factors**	**Opportunities**
EAAB articulation with inter-sectoral policies and initiatives that directly or indirectly support IYCF	The execution of inter-sectoral policies and the integration of education, agriculture, and social assistance in early childhood are important to indirectly support the EAAB; The articulation of the coordination of the EAAB with the Infant Education sector allows developing IYCF activities with kindergarten workers.
EAAB articulation with BF supportive sectoral policies and programs	Infant health policies and programs benefited the EAAB actions because they also aimed to improve IYCF; A state law that promotes IYCF supported scaling up of the EAAB; A municipal program to support BF enhanced the communication between the delivery hospitals and the PHC units that implemented EAAB.
Development of research on IYCF and policy implementation analysis	The strong scientific evidence supporting IYCF motivates the PHC coordinators to implement the EAAB; Research promoting the use of the EAAB Tutor Support Manual improved tutors’ performance and resulted in positive changes in the activities of PHC units.

Source: Produced by the authors. Notes: BF (breastfeeding); CF (complementary feeding); EAAB (Brazilian Breastfeeding and Complementary Feeding Strategy (Estratégia Amamenta e Alimenta Brasil)); ENPACS (National Strategy for Promotion of Complementary Healthy Eating (Estratégia Nacional para Alimentação Complementar Saudável)); FAN (Financing Food and Nutrition Actions (Financiamento das Ações de Alimentação e Nutrição)); IYCF (Infant and Young Child Feeding); MoH (Ministry of Health); PHC (Primary Health Care); RAB (Brazilian Breastfeeding Network (Rede Amamenta Brasil)).

**Table 4:** Motivating needs, Core Functions, and Forms for the implementation of the EAAB.

Motivating Needs	Core Functions	Forms
Coordination	Presence of coordinators at the three levels of government and in the inter-federative and intra-sectoral articulation	The MoH, the states, and the municipalities appoint professionals from the Health Secretariats to coordinate the EAAB. Professionals from technical areas of Health Secretariats (Food and Nutrition and Child Health) work together at the three government levels to coordinate the EAAB. The municipal Health Secretariats appoint professionals with experience in continuing learning or in the implementation of the Brazilian programs RAB or ENPACS to coordinate the EAAB.
Funding and resources	Resource allocation for the implementation of the EAAB	The MoH transfers resources to states and municipalities for the implementation of the EAAB. The MoH transfers resources to states and municipalities through the Food and Nutrition Fund (FAN). States and municipalities use FAN resources for the implementation of the EAAB. States and municipalities allocate their own resources for the implementation of the EAAB.
Scaling up the EAAB	Implementation planning across the governmental levels	The MoH makes agreements with states and municipalities to organize the implementation of the EAAB. States select priority regions/municipalities for the implementation of the EAAB based on the nutritional status of children under 2 years old. Municipalities prepare an EAAB implementation plan and define the priority territories/PHC units for the implementation of the EAAB based on the nutritional status of children under 2 years old. Municipal coordinators make inter-sectoral articulations to develop more training workshops. Municipal coordinators support tutors’ agenda to develop training workshops in PHC units.
Improvement of professional skills	Establishing a group of national trainers, and training of tutors, and PHC workers	The MoH provides the EAAB Implementation Guide, offers theoretical support materials (e.g., food guide for Brazilian children, the EAAB online course, and the Tutors Support Manual), and establishes a group of national trainers. State coordinators organize the infrastructure and provide human and material resources for the first workshop and the complementary training workshops. State coordinators indicate professionals to work as state trainers to lead more tutor training workshops. National and state trainers lead the tutor training workshops in the state regionals of health and municipalities. Municipal coordinators select professionals to work as tutors and request more tutor training workshops if needed. Municipal coordinators and tutors provide material resources for the development of training workshops in PHC units. PHC workers and managers arrange their schedules to join the training workshop in the PHC unit. Tutors and PHC workers plan a schedule for developing complementary workshops and the EAAB action plan. Tutors follow up on at least one PHC unit and give support to the action plan development. Tutors use the Tutor Support Manual to guide the complementary workshops.
Improvement of the work process	Development of activities to obtain the certification	PHC workers systematically develop actions, individually or in groups, to promote IYCF. PHC workers accomplish at least one activity from the action plan. PHC workers record the indicators of IYCF (exclusive BF in children under 6 months old, and CF in children from 6 to 24 months old) in the monitoring system data. PHC workers use a protocol to organize children’s health care. PHC workers comply with NBCAL and Law 11.265/06 and do not distribute milk or infant formulas in PHC units, except for special cases established by law, ordinance, or decree.
Impacts on population and implementation sustainability	Implementation monitoring and evaluation	The MoH provides and maintains systems for monitoring the implementation of the EAAB and the IYCF indicators. The MoH systematically monitors and evaluates the implementation of the EAAB. The MoH consults the EAAB trainers and tutors through national surveys and workshops to monitor the implementation quality. State and municipal coordinators monitor the implementation of the EAAB via the system and support tutors’ performance through meetings, events, and other technologies available. Municipal coordinators use supplementary monitoring strategies to control the EAAB monitoring. Municipal and state coordinators provide technical support to tutors and PHC workers on how to use the systems properly. Tutors enter information about the participation of PHC workers in the workshops and their action plan development into the EAAB monitoring system. PHC workers enter data on the IYCF indicators in the system.
Social Communication	Dissemination of the EAAB	The MoH publishes the results of the implementation of the EAAB. The MoH provides implementation certification to the PHC units that fulfill the certification criteria. State coordinators host events to promote the EAAB and share experiences about the implementation of the EAAB. Municipal coordinators promote the EAAB in events and in the Health Municipal Committees.

Source: Developed by the authors with data obtained from document review, and interviews with key informants. Notes: BF (breastfeeding); CF (complementary feeding); EAAB (Brazilian Breastfeeding and Complementary Feeding Strategy (Estratégia Amamenta e Alimenta Brasil)); ENPACS (National Strategy for Promotion of Complementary Healthy Eating (Estratégia Nacional para Alimentação Complementar Saudável)); FAN (Financing of Food and Nutrition Actions (Financiamento das Ações de Alimentação e Nutrição)); IYCF (Infant and Young Child Feeding); MoH (Ministry of Health); NBCAL (Brazilian Norm for the Commercialization of Food for Infants and Young Children, Pacifiers and Baby Bottles (Norma Brasileira de Comercialização de Alimentos para Lactentes e Crianças de Primeira Infância, Bicos, Chupetas e Mamadeiras)); PHC (Primary Health Care); RAB (Brazilian Breastfeeding Network (Rede Amamenta Brasil)).

## Supplementary Materials

The following supporting information can be downloaded at: https://www.mdpi.com/article/10.3390/ijerph19169839/s1, Table S1: Description of Brazilian Breastfeeding and Complementary Feeding Strategy (Estratégia Amamenta e Alimenta Brasil—EAAB) according to the Template for Intervention Description and Replication (TIDieR), Figure S1: Initial PIP diagram of the EAAB implementation.
