# Supplementary material for: Brazilian Strategy for Breastfeeding and Complementary Feeding Promotion: A Program Impact Pathway Analysis
Source: Int J Environ Res Public Health. 2022 Aug 10;19(16):9839. doi: 10.3390/ijerph19169839 (PMC9408563; doi:10.3390/ijerph19169839)
Supplement: Supplementary file 1 [file ijerph-19-09839-s001.zip › ijerph-1818179-supplementary.pdf]

Supplementary Materials

Table S1 -Description of Brazilian Breastfeeding and Complementary Feeding Strategy (Estratégia Amamenta e Alimenta Brasil—EAAB) according to the Template for Intervention Description and Replication (TIDieR).

**NAME**	The Brazilian Breastfeeding and Complementary Feeding Strategy (Estratégia Amamenta e Alimenta Brasil—EAAB) aims to improve BF rates and healthy CF in Brazil by training primary health care workers.
**WHY**	BF and healthy CF are essential for children's nutrition and health development. Counseling skills are important for promoting IYCF in public health care services. The EAAB ultimately improves infant nutrition care by training health workers in PHC units, so they can develop activities to promote healthy feeding practices among children under 2 years old.
**WHAT**	The EAAB includes training workshops based on critical and reflective thinking methodology and proposes continuing learning workshops. The MoH establishes a group of national trainers, who are responsible for leading tutor training workshops in states and municipalities. Tutor training workshops are 32-hour in-person meetings divided into four days of activities.<br>In municipalities, tutors work as coaches in PHC units and teach about BF and nutrition counseling strategies and how health care workers can develop an action plan. The first training workshop in each PHC unit is a 4-hour in-person meeting. Complementary workshops can be scheduled.<br>The EAAB offers a complementary online course to train primary health care workers in BF and healthy CF.<br>The EAAB implementation certification is given by the MoH to the PCH units that:<br>1 — develop systematic actions, individually or in groups, to promote BF and healthy CF;<br>2 —monitor the BF and CF rates using the current system;<br>3 — have a protocol to help organize child health care and detect problems related to BF and CF;<br>4 — comply with the NBCAL and Law 11.265/2006 and do not distribute "substitutes" for breast milk in the PHC units;<br>5 — have at least 85% of its workers participating in the workshops;<br>6 — carry out at least one incentive action for BF and one for healthy CF agreed in the action plan.
**WHO PROVIDED**	**Coordinators at national, state, and municipal levels:** professionals from Health Secretariats that are responsible for planning, funding, organizing, and monitoring the EAAB implementation.<br>**National and State Trainers:** professionals with higher education that are responsible for training the EAAB tutors in states and municipalities.

	**Tutors:** professionals with higher education that are responsible for training health workers in PHC units. Tutors are supposed to support teams of workers in planning EAAB actions to improve BF and CF practices and to obtain the implementation certification.<br><br>**PHC workers:** health care workers responsible for developing and monitoring the EAAB actions to promote BF and healthy CF with mothers and caregivers.
**HOW**	The actions to promote BF and healthy CF with mothers and caregivers can be adapted to local contexts and include: face-to-face individual activities, such as counseling; collective activities, such as the formation of educational groups; and remote activities, such as guidance through WhatsApp groups.
**WHERE**	The EAAB is implemented in PHC units in all states of Brazil. Activities to promote IYCF take place mostly in PHC units, but it is also recommended to develop actions in collaboration with intersectoral organizations, such as kindergartens and social assistance centers.
**MODIFICATIONS**	The MoH proposed some modifications to the EAAB implementation design in 2020, including 1) offering an online course to train tutors; 2) reducing the workload of the face-to-face tutor training workshop from 32 to 16 hours; and 3) providing expert staff to support state and municipal coordinators in planning the EAAB implementation.
**HOW WELL (planned)**	Training of tutors and capacity building of PHC units teams: It is planned to train EAAB tutors in all Brazilian municipalities. Monitoring is done through a specific EAAB management system. It monitors the number of training workshops held, PHC units' action plans, and their compliance with the certification criteria. Tutors are responsible for entering this information into the system, and the federal, state, and municipal coordinators are responsible for monitoring the data.<br><br>Quality improvement of IYCF activities and impact on child nutrition: it is planned that the actions developed by the PHC units teams, agreed upon in the EAAB action plan, will improve the quality of BF and CF promotion and positively impact the food consumption indicators of children under two years of age. The data on the actions to promote BF and CF carried out with the population are recorded by the PHC workers in the Health Information System for Primary Care. The System for Food and Nutrition Surveillance (Sisvan) is used by the PHC workers to record data on BF, infant food intake, and infant nutritional status. The federal, state, and municipal coordinators are responsible for monitoring the data.
**HOW WELL (current)**	Training of tutors and capacity building of PHC units teams: By 2020, a total of 6,348 tutors were trained. The tutors conducted 3627 workshops in PHC units and taught 56,625 health workers. A total of 189 PHC units have been certified (data from the MoH in 2020).<br><br>Quality improvement of IYCF activities and impact on child nutrition: The national data coverage on child food consumption in the Sisvan system was 5% in 2020. The prevalence of the consumption of ultra-processed foods by children under 23 months of age was 45%, and the prevalence of exclusive BF on children under 6 months was 55.8%.

Source: Developed by the authors, adapted from Hoffmann et al. [15] and Ministry of Health of Brazil [16,28,29]. **Notes**: BF (breastfeeding); CF (complementary feeding); EAAB) Brazilian Breastfeeding and Complementary Feeding Strategy (Estratégia Amamenta e Alimenta Brasil)); IYCF (Infant and Young Child Feeding); MoH — (Ministry of Health); NBCAL (Brazilian Norm for the Commercialization of Food for Infants and Young Children, Pacifiers and Baby

Bottles (Norma Brasileira de Comercialização de Alimentos para Lactentes e Crianças de 1ª Infância, Bicos, Chupetas e Mamadeiras)); PHC (Primary Health Care).

Figure S1 - Initial PIP diagram of the EAAB implementation

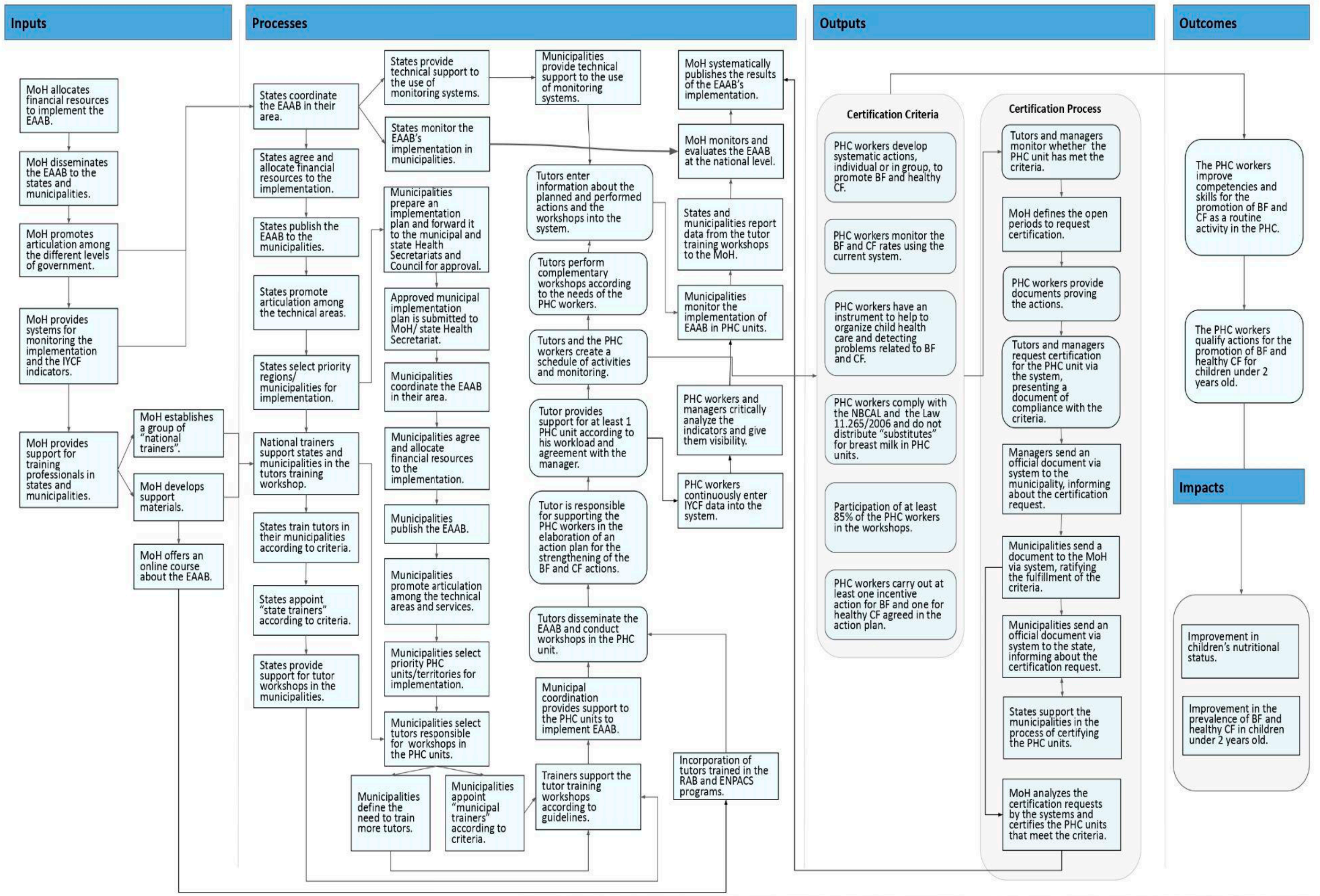

Source: Developed by the authors with data obtained from the document review [16,28,29]. **Notes**: BF (breastfeeding); CF (complementary feeding); EAAB (Brazilian Breastfeeding and Complementary Feeding Strategy (Estratégia Amamenta e Alimenta Brasil)); ENPACS (National Strategy for Promotion of Complementary Healthy Eating (Estratégia Nacional para Alimentação Complementar Saudável));IYCF (Infant and Young Child Feeding); MoH (Ministry of Health); NBCAL (Brazilian Norm for the Commercialization of Food for Infants and Young Children, Pacifiers, and Baby Bottles (Norma Brasileira de Comercialização de Alimentos para Lactentes e Crianças de 1ª Infância, Bicos, Chupetas e Mamadeiras)); PHC (Primary Health Care); RAB (Brazilian Breastfeeding Network (Rede Amamenta Brasil)). **Square boxes:** structural activities of the EAAB implementation; **Rounded boxes:** activities at the PHC units level.
